# Survival Impact of Aggressive Treatment and PD-L1 Expression in Oligometastatic NSCLC

**DOI:** 10.3390/curroncol28010059

**Published:** 2021-01-20

**Authors:** Camille Gauvin, Vimal Krishnan, Imane Kaci, Danh Tran-Thanh, Karine Bédard, Roula Albadine, Charles Leduc, Louis Gaboury, Normand Blais, Mustapha Tehfe, Bertrand Routy, Marie Florescu

**Affiliations:** 1 Department of Internal Medicine, University of Montreal, Montreal, QC H3T 1J4, Canada; camille.gauvin@umontreal.ca; 2 Department of Pathology and Cell Biology, University of Montreal, Montreal, QC H3T 1J4, Canada; vimal.krishnan@umontreal.ca (V.K.); imane.kaci@umontreal.ca (I.K.); danh.tran-thanh.chum@ssss.gouv.qc.ca (D.T.-T.); karine.bedard.chum@ssss.gouv.qc.ca (K.B.); roula.albadine.chum@ssss.gouv.qc.ca (R.A.); 3 Institute for Research in Immunology and Cancer (IRIC), Montreal, QC H3T 1J4, Canada; louis.gaboury@umontreal.ca; 4 Department of Pathology, Centre Hospitalier de l’Université de Montréal (CHUM), Montreal, QC H2X 0C1, Canada; charles.leduc.chum@ssss.gouv.qc.ca; 5 Centre de Recherche du Centre Hospitalier de l’Université de Montréal (CRCHUM), Montreal, QC H2X 0A9, Canada; normand.blais.chum@ssss.gouv.qc.ca (N.B.); mustapha.tehfe.chum@ssss.gouv.qc.ca (M.T.); bertrand.routy@umontreal.ca (B.R.); 6 Service of Molecular Biology, Centre Hospitalier de l’Université de Montréal (CHUM), Montreal, QC H2X 0C1, Canada; 7 Hematology-Oncology Division, Department of Medicine, Centre Hospitalier de l’Université de Montréal (CHUM), Montreal, QC H2X 0C1, Canada

**Keywords:** non-small cell lung cancer, oligometastatic, overall survival, PD-L1, aggressive treatment, immune checkpoint inhibitors, immunotherapy, prognostic factor

## Abstract

Background: Studies have shown that aggressive treatment of non-small cell lung cancer (NSCLC) with oligometastatic disease improves the overall survival (OS) compared to a palliative approach and some immunotherapy checkpoint inhibitors, such as anti-programmed cell death ligand 1 (PD-L1), anti-programmed cell death protein 1 (PD-1), and T-Lymphocyte-associated antigen 4 (CTLA-4) inhibitors are now part of the standard of care for advanced NSCLC. However, the prognostic impact of PD-L1 expression in the oligometastatic setting remains unknown. Methods: Patients with oligometastatic NSCLC were identified from the patient database of the Centre hospitalier de l’Université de Montréal (CHUM). “Oligometastatic disease” definition chosen is one synchronous metastasis based on the M1b staging of the eight IASLC (The International Association for the Study of Lung Cancer) Classification (within sixth months of diagnosis) or up to three cerebral metastasis based on the methodology of the previous major phase II randomized study of Gomez et al. We compared the OS between patients receiving aggressive treatment at both metastatic and primary sites (Group A) and patients receiving non-aggressive treatment (Group B). Subgroup analysis was performed using tumor PD-L1 expression. Results: Among 643 metastatic NSCLC patients, we identified 67 patients with oligometastasis (10%). Median follow-up was 13.3 months. Twenty-nine patients (43%) received radical treatment at metastatic and primary sites (Group A), and 38 patients (57%) received non-aggressive treatment (Group B). The median OS (mOS) of Group A was significantly longer than for Group B (26 months vs. 5 months, *p* = 0.0001). Median progression-free survival (mPFS) of Group A was superior than Group B (17.5 months vs. 3.4 months, *p* = 0.0001). This difference was still significant when controlled for primary tumor staging: stage I (*p* = 0.316), stage II (*p* = 0.024), and stage III (*p* = 0.001). In the cohort of patients who were not treated with PD-L1 inhibitors, PD-L1 expression negatively correlated with mOS. Conclusions: Aggressive treatments of oligometastatic NSCLC significantly improve mOS and mPFS compared to a more palliative approach. PD-L1 expression is a negative prognostic factor which suggests a possible role for immunotherapy in this setting.

## 1. Introduction 

Recent classification by the International Association for the Study of Lung Cancer (IASLC) differentiates non-small cell lung cancer (NSCLC) with only one metastasis (M1b, stage IVA) from plurimetastatic (M1c). The M1b stage is defined in this classification as one metastasis limited to a single organ. The median overall survival (mOS) of stage M1b patients (11.4 months) is similar to that of stage M1a patients, having one metastasis to the contralateral lung (11.8 months), whereas the mOS for patients with multiple lesions in one other organ in addition to the primary site (7.0 months) is more similar to that of plurimetastatic patients (6.2 months) [1]. There is some variation in the literature regarding the definition of oligometastatic disease. Some studies include up to five brain metastases if limited to one site, with possible localized treatment [2]. The European Organization of Research and Treatment of Cancer (EORTC) consensus states that oligometastatic disease consists of up to five metastasis and three organs [3]. As highlighted in a Canadian consensus, the maximum number of metastatic sites remains variable but ranges from three or fewer to five or fewer and up to six extracranial lesions [4]. The latest IASLC classification presents a need to better define or understand management of NSCLC in the context of oligometastatic disease.

Analyses of NSCLC metastatic patients of all sites showed that between 25% and 50% of patients developing metastatic disease had three or fewer metastatic sites [5,6]. Prospective data suggest that a subset of oligometastatic NSCLC treated aggressively may achieve long-term survival compared with conventional chemotherapy, with progression-free survival (mPFS) improvement from 4 months to a median PFS (mPFS) of 12.1 months and overall survival (mOS) from 7.9 months to 13.5 months [7]. Another prospective study from a randomized phase II trial of stage IV NSCLC showed significant mPFS (14.2 months vs. 4.4 months, *p* = 0.022) and mOS (41.2 months vs. 17 months, *p* = 0.017) benefits with aggressive local treatment compared with systemic standard therapy [8]. Furthermore, retrospective data showed that selected patients with stage IV cerebral oligometastatic NSCLC may have survival benefit after treatment of both primary and metastatic sites [9]. Among this population, N0–N1 patients may be surgical candidates for this approach [9].

The therapeutic options for the definitive treatment of metastatic NSCLC depend on the site of metastatic disease. Options include surgery, ablative stereotactic radiotherapy (RT), microwave ablative RT and radiofrequency ablative RT. Despite progress and development of these aggressive therapeutic approaches, surgeons still retain some reservations on performing surgery at the metastatic site in this clinical context. Retrospective data have shown that the best prognostic factors consist of the definitive treatment of the primary site, nodal status, and an interval without progression of 6 to 12 months [10]. Among these prognostic factors is also the chronological occurrence of the metastasis. Synchronous metastases, which by definition appears within six months following diagnosis, are associated with a worse survival than asynchronous metastases, which appears beyond six months [11]. To date, research has mostly addressed cerebral metastasis treatment and justifies the need to study all NSCLC metastatic sites.

Immunotherapy with anti-PD-1/PD-L1 agents improve long-term survival in advanced NSCLC and is now considered a standard treatment either alone or with chemotherapy for metastatic NSCLC since 2016 [12]. While tumor cell anti-programmed cell death ligand 1 (PD-L1) expression directs selection for immunotherapy, it has not been characterized in NSCLC patients with oligometastatic disease.

In this retrospective study, we aimed to determine the survival benefit of patients with oligometastatic NSCLC who received aggressive treatment compared to those who received palliative treatment in our center. Our study also aimed to better characterize oligometastatic NSCLC and to determine the incidence and prognostic value of PD-L1 expression in the NSCLC oligometastatic population, which is yet to be determined in this setting. 

## 2. Materials

### 2.1. Study Design

This single-center retrospective study was based on NSCLC patients diagnosed at the Centre Hospitalier de l’Université de Montréal (CHUM) between 2005 and 2015. Databases used for this study included the CHUM electronic files and the “Système d’archivage de données oncologiques” (SARDO) neoplastic data register. Ethical approval was obtained by the local institutional review board.

### 2.2. Patients and Pathology Specimens

The following are inclusion criteria for the study population: a pathologically proven diagnosis of NSCLC; an available Tumor Node and Metastasis (TNM) Staging; one synchronous metastasis based on the M1b staging of the 8th IASLC Classification [1]. (within 6 months of diagnosis) or up to three cerebral metastases based on the methodology of the previous major phase II randomized study of Gomez et al. [8]; a minimum follow-up period of 6 months; and no more than one contralateral lung metastasis.

The selected 67 patients were ranked according to their “modified” staging, which refers to the combined tumoral (T) stage and nodal (N) stage without metastasis (M) stage of the IASLC staging 8th edition. These are defined as follow: Stage I: T1–2 N0 M1b, Stage II: T1–2 N1 M1b and T3–4 N0 M1b, and Stage III: T1–2 N2 and T3–4 N1 M1b. This modification explains the differences between our groups when staging was according to nodal status (N0, N1, N2, and N3) or according to stages I, II, and III. For example, certain stage II and III patients now became N0 and N1.

For patients without Epidermal growth factor receptor (EGFR) or Anaplastic lymphoma kinase (ALK) status at baseline, DNA was extracted from tissue specimens when possible using the QIAamp DNA FFPE Tissue Kit (QIAGEN, Toronto, ON, Canada). EGFR mutational analysis (exons 18, 19, 20, and 21) was performed using the EGFR Mutation Analysis Kit (EntroGen, Woodland Hills, CA, USA) and real-time PCR results were analyzed using LightCycler 480 (Roche Diagnostics, Laval, QC, Canada). ALK immunostaining was performed with D5F3 antibody (Roche Diagnostics, Indianopolis, IN, USA), and interpretation was confirmed by a pathologist.

For PD-L1 analysis, 39 patients had available tissue blocks with sections containing at least 100 viable tumor cells. Samples were stained with SP263 antibody (Roche Diagnostics, Indianopolis, IN, USA) against PD-L1. Membrane staining of ≥25% of tumor cells was considered positive. We used this cut-off value because the test used was created based on its ability to identify responders found with this value in a Phase 1/2 Study of durvalumab at the moment the analysis was done in 2016, supporting its use for the evaluation of NSCLC and Neck squamous cell carcinoma (HNSCC) [13,14]. In 10 cases, both primary and metastatic tissues were available for correlation. No patients in this analysis had received immunotherapy.

### 2.3. Treatments

Treatments were categorized as either curative or palliative. Herein, the term “aggressive treatment” refers to treatment with curative intent. Curative intent treatments included surgical resection, external Radion therapy (RT) (60 Grays {Gy}administered in 30 fractions {Fx}), cyberknife, volume-modulated arctherapy (VMAT) (60 Gy/3 Fx), and concomitant chemoradiation therapy at the site of primary disease and mediastinal nodal disease if present. For brain lesions, neurosurgery +/− adjuvant RT as well as cyberknife were considered curative treatments. For bone, hepatic, and adrenal lesions, surgical resection with negative margins were considered curative. Palliative treatments included the following: RT of 30 Gy/10 Fx or 20 Gy/5 Fx at the primary site, whole brain RT of 20 Gy/5 Fx, RT of 20 Gy/5 Fx or 8 Gy/1 Fx at bone site as well as chemotherapy (cisplatin-pemetrexed, cisplatin-gemcitabine, carboplatin-taxol, carboplatin-pemetrexed), tyrosine-kinase inhibitor (Gefitinib), and routine palliative symptomatic care as needed.

Patients were divided into two groups based on treatment type. Group A included patients who received aggressive treatments at both metastatic and primary sites which were defined by treatment for curative intent at both sites. Two subgroups were included: aggressive treatment at both sites with adjuvant chemotherapy (A1) and aggressive treatment without adjuvant chemotherapy (A2). Group B represented patients who received non-aggressive treatments, which could involve aggressive treatment of either the primary (B1) or metastatic site (B2), but not both. Palliative approaches, including patients who did not receive any treatments, were included in Group B (B3).

### 2.4. End Points

The main outcome was to compare the OS between oligometastatic NSCLC patients who received aggressive treatment at both primary and metastatic sites (Group A) and those who received non-aggressive treatment (Group B), divided by stage. We then examined prognostic factors including PD-L1 expression and their correlation between the primary and metastatic sites.

### 2.5. Statistical Analysis

Survival rates were calculated based on the Kaplan-Meier method. Association between variables were tested using the Chi-Square test and Cox regression. SPSS Statistics 24 (IBM, Markham, ON, Canada) and GraphPad Prism version 7 (GraphPad Software, San Diego, CA, USA) were used.

## 3. Results

### 3.1. Oligometastatic Non-Small Cell Lung Cancer (NSCLC) Prevalence

**Figure 1:**
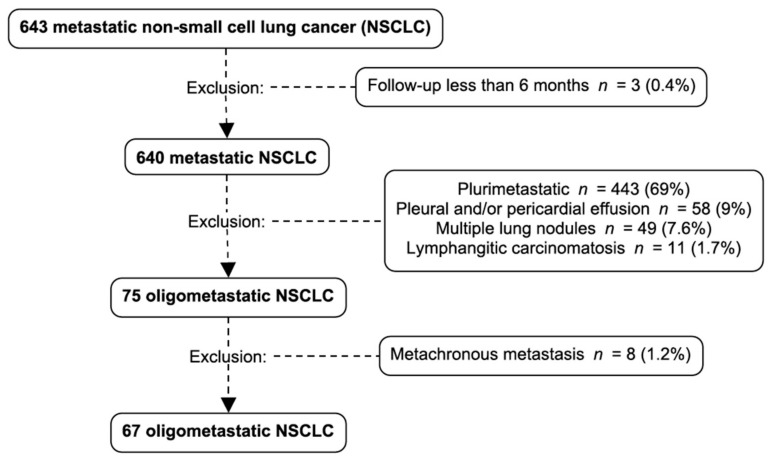
Patient selection and exclusion criteria: 643 metastatic non-small cell lung cancer patients were identified. Three patients were excluded because of a follow-up shorter than 6 months, 443 patients were excluded because they were plurimetastatic at diagnosis, 58 were excluded because of a pleural or pericardial effusion, 49 because they had multiple lung nodules and 11 because of the presence of lymphangitic carcinomatosis. Among the 75 oligometastatic NSCLC, 8 were excluded because of metachronous metastasis. NSCLC: non-small cell lung cancer.

Of the 801 NSCLC patients reviewed from our databases, 643 of them had adequate documentation. We identified 67 oligometastatic patients for our study. They represented 10.4% of metastatic NSCLC with sufficient data who were oligometastatic at diagnosis (Figure 1). The median follow-up duration was 13 months.

### 3.2. Metastatic Sites Prevalence 

The brain was the most common metastatic site. Among 67 patients, 37 (55%) had a single cerebral metastasis, 9 (13%) had two cerebral metastases and 4 (6%) had three cerebral metastases. The second most prevalent metastatic site of our cohort was the adrenal gland with six (9%) patients presenting one metastasis, followed by five (7%), 4 (6%), and two (3%) patients respectively with a single contralateral lung, bone, and hepatic metastasis. The median overall survival of the patients with brain metastasis (*n* = 50) based on the number of metastasis were respectively 17 months, 8 months, and 3 months for patients presenting with one, two, and three metastasis (*p* = 0.121) (Figure S1).

### 3.3. Treatments

Patient demographic and clinical characteristics are presented in Table 1 and were divided by treatment type in Table S1. Staging classified by metastatic sites are presented in Table 2. Of the 67 patients who met the eligibility of oligometastatic disease, 29 (43%) were in group A, treated aggressively at both primary and metastatic sites and 38 (57%) were included in group B, not treated aggressively at both sites. We identified 52 of 67 patients (78%) treated with aggressive intent at one or both sites (A1, A2, B1, or B2). More specifically, 33% of patients received aggressive treatment at both the primary and metastatic sites and 10% received aggressive treatment at both the primary neoplastic and metastatic site with adjuvant chemotherapy. A total of 18% had aggressive treatment of the metastasis only; 10% received aggressive treatment of the metastasis and chemotherapy +/− palliative RT; and 10% received aggressive treatment at both the primary neoplastic and metastatic site, with adjuvant chemotherapy. The majority of patients with cerebral metastasis (92%) and contralateral lobe metastasis (80%) received aggressive treatments. In contrast, the majority (83.3%) of patients with adrenal metastasis received palliation, including two patients who were under observation only.

We performed Cox regression analysis for the Eastern Cooperative Oncology Group (ECOG) score at the time of diagnosis and age. These factors did not have a statistically significant impact on survival outcome, confirming that the only impact on survival was the treatment approach: aggressive at both sites compared with other approaches.

#### 3.3.1. Survival of Patients with Aggressive Treatment at Both Metastatic and Primary Sites Compared to Non-Aggressive Treatments

**Figure 2:**
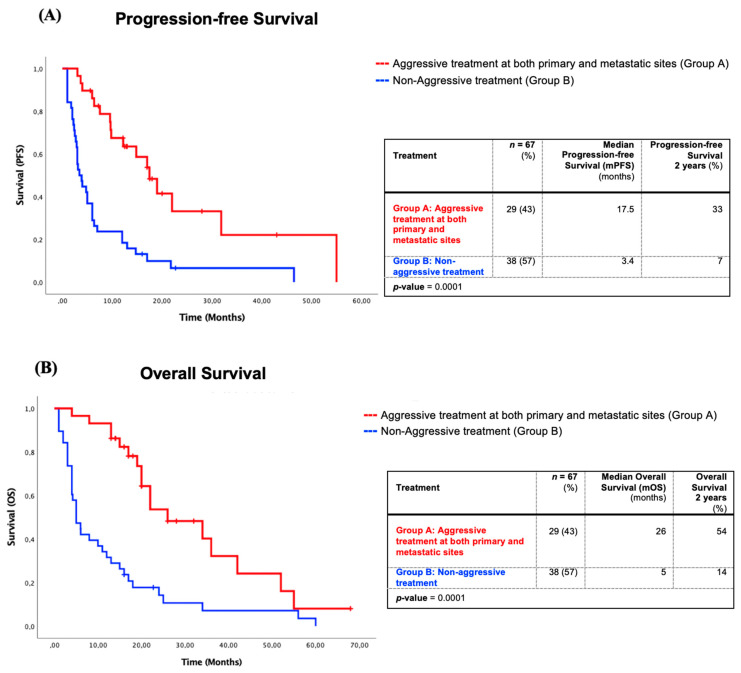
Survival of patients with aggressive treatments at both primary and metastatic sites (Group A) in comparison with non-aggressive treatments (Group B). (**A**) Kaplan–Meier progression-free survival analysis of patients with aggressive treatments at both primary and metastatic sites (Group A) in comparison with non-aggressive treatments (Group B). (**B**) Kaplan–Meier overall survival analysis of patients with aggressive treatments at both primary and metastatic sites (Group A) in comparison with non-aggressive treatments (Group B).

The mPFS of aggressive treatment Group A was 17.5 months compared to 3.4 months of non-aggressive treatment Group B (Figure 2). The mPFS at 1, 2, and 5 years for both groups were respectively at 68%, 33%, and 0% vs. 18%, 7%, and 0% (*p* = 0.0001). The mOS of Group A also showed a statistically significant advantage of 26 months compared to 5 months of Group B (Figure 2). The OS at 1, 2, and 5 years for both groups were respectively at 93%, 54%, and 8% vs. 32%, 14%, and 0% (*p* = 0.0001).

#### 3.3.2. Survival Impact of Four Management Approaches in Oligometastatic NSCLC

We also compared three different aggressive treatment groups and one palliative approach group (Figure S2): Aggressive treatment at both metastatic and primary sites (Group A); radical treatment to the primary site only (Group B1), radical treatment to the metastatic site only (Group B2); and palliative treatment (Group B3). The mOS of groups A and B1 was statistically significantly greater at 26 and 24 months when comparing independently the four groups (*p* = 0.0001). The mPFS was also statistically significantly greater when comparing independently between the four treatment groups for these two groups with 17 months for group A receiving radical treatment at both sites and 22 months for group B1 who received radical treatment to the primary tumor site only (*p* = 0.0001). The patients who received radical treatment to the metastasis only (Group B2) and palliative treatment (Group B3) had a mOS of five and three months and a mPFS of four and three months, respectively.

#### 3.3.3. Overall Survival (OS) and Progression-Free Survival (PFS) Analyzed by “Modified” Stage of Patients with Aggressive Treatment at Both Sites versus Other Treatments

- Stage I: The mOS for the “modified” stage I oligometastatic NSCLC, which refers to the combined stage T and N of the IASLC staging eighth edition, radically treated at both sites (Group A) was 42 months compared with 16 months for other forms of treatments (Group B) (*p* = 0.316). The mPFS was the same in both group (*p* = 0.669) (Table S2).
- Stage II: The mOS of “modified” stage II of oligometastatic NSCLC patients of Group A was 34 months vs. 6 months for group B and was statistically significant (*p* = 0.024). The mPFS of the “modified” stage II patients treated radically at both sites was not reached vs. 6.0 months of group B patients (*p* = 0.016) (Table S2).
- Stage III: The mOS and mPFS for “modified” stage III was significantly higher in Group A with an mOS of 22 months vs. 4 months for Group B (*p* = 0.001). The mPFS of the “modified” stage III of Groups A and B were respectively 19 months vs. 3 months (*p* = 0.003) (Table S2).

### 3.4. Epidermal Growth Factor Receptor (EGFR) Mutation Status and Anaplastic Lymphoma Kinase (ALK) Expression Profile 

Forty-one patients had a documented EGFR status or had sufficient tissue for EGFR mutation analysis (exon 18, 19, 20, and 21). None had a detectable mutation. Forty-nine patients had a documented ALK status or had sufficient tissue for ALK immunohistochemistry, and no overexpression was detected (Table 1). However, due to insufficient tissue for analysis, the status of EGFR and ALK was unknown in 39% and 27% of the patients.

### 3.5. Programmed Cell Death Ligand 1 (PD-L1) Expression Profile 

Thirty-nine patients had available tissue (either primary or metastatic) for PD-L1 immunohistochemistry (Table 1 and Table S3). Overall, PD-L1 positivity was 44%. When stratified by specimen type, positivity in lung primary specimens was 55% (*n* = 22) and in metastatic tissue, 30% (*n* = 27).

A subgroup of ten patients had both primary and metastatic tissue available for analysis (Table S4). Correlation of PD-L1 status between primary and metastatic site in these patients was 80% (8/10). The two discordant cases were noted to have heterogenous staining patterns, which could explain the discordance.

### 3.6. PD-L1 Status and Clinical Outcomes 

No clinical (sex, age, ECOG, Tumor Node and Metastasis Staging (TNM), and smoking history) and pathological (histology and metastatic site) characteristics at baseline were significantly associated with PD-L1 status (Table 3).

**Figure 3:**
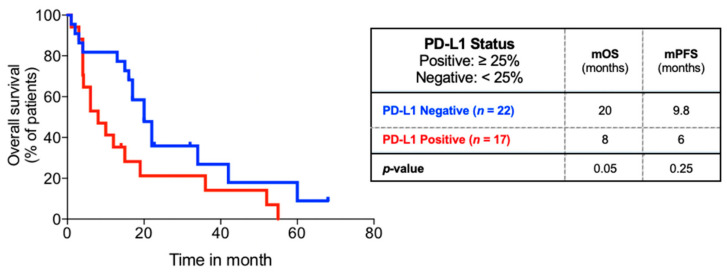
Overall survival of subgroup of 39 patients comparing PD-L1 status. Kaplan–Meier overall survival analysis of subgroup of patients with PD-L1 status available comparing PD-L1 status.

In our cohort of 39 patients, none had received immunotherapy, 59% (23/39) of patients received aggressive treatment for the primary tumor, and 86% (31/39) received aggressive treatment for metastatic brain disease. The mPFS was not significantly different between PD-L1 status groups (14.7 months vs. 6.0 months, *p* = 0.14). The mOS was significantly shorter for PD-L1 positive patients compared to PD-L1 negative patients (8 months vs. 20 months, *p* = 0.05) (Figure 3). This association was independent of aggressive treatment of the primary site (Figure S3), but not independent of aggressive metastatic disease treatment (Figure S4).

## 4. Discussion

Our study demonstrates a significantly longer mOS and mPFS for patients who received radical treatment at both primary and metastatic sites compared with palliative treatments (mOS: 26 months vs. 5 months, *p* = 0.0001; and mPFS: 17.5 months vs. 3.4 months, *p* = 0.0001). This represents at least two advantages of an aggressive treatment approach for oligometastatic NSCLC patients. First, it represents one year without any treatment, which is an important quality of life factor for oncology patients. Second, even after progression, this group had almost one more year of survival compared with approximately five months if treated otherwise. 

Our data showed no evidence of mOS benefit for treatment addressing only the metastatic site when compared to the palliative approach group (five months vs. three months). Aggressive treatment at both sites or at primary site only were the two approaches associated with better mOS and mPFS, at 17 and 22 months, respectively, without progression.

For all NSCLC stages, an aggressive treatment at both primary and metastatic sites was associated with better mOS compared to other treatments, which is concordant with prospective studies [8]. When stratified by “modified” stage, which is the T and N stage combined without the M Stage of the IASLC staging eighth edition, this benefit remained significant for stages II (T1–2 N1 M1b and T3–4 N0 M1b) and III (T1–2 N2 and T3–4 N1 M1b), but not for stage I.

PD-L1 expression by tumor cells is an important mechanism of immune evasion and is a marker for selection of immunotherapy. Here, we show that PD-L1 positivity is a negative prognostic factor for oligometastatic NSCLC. This association appears to be preserved when controlling for treatment aggressivity, which makes the prospect of immunotherapy particularly interesting for these patients. In a systematic review by Brody and colleagues, four studies similarly reported reduced OS for PD-L1 positive NSCLC patients [15]. Thus far, molecular profiles as prognostic factors have not been studied in oligometastatic disease [16]. PD-L1 appears to be an independent prognostic factor with no association with clinical or pathological characteristics in our study.

The expression profile of PD-L1 in primary malignant tissue versus metastatic tissue has been seldom reported in the literature. A study by Uruga et al. reported a discrepancy of 38% in primary lung adenocarcinomas versus nodal metastases [17]. A study on triple negative breast carcinomas and paired nodal metastases reported a 20% discrepancy [18], and a similar study for renal cell carcinoma reported a 31% discrepancy [19]. Our study is the first one to examine this issue in the context of oligometastatic NSCLC. In our limited sub-group of patients, PD-L1 concordance between the primary pulmonary tissue and metastatic tissue was 80% (8/10). Tumor heterogeneity may have impacted the assessment of the two discordant cases. This interesting finding has potential therapeutic implications, as metastatic tissue appears to preserve the same PD-L1 status in oligometastatic patients. The cut-off choice of 25% for positive immunohistochemistry staining in our study is a limitation because it is not the current cut-off used, but it was the cut-off value recommended for the SP263 antibody (Ventana) against PD-L1 in 2016 [13,14], when the pathological analysis was done. However, SP263 has since been approved for use with pembrolizumab and nivolumab, and the positivity cut-offs have been revised to 1% and 50% [20]. We acknowledge that, at the time of publication, this discrepancy may hinder translation of our data to clinical practice, namely for patients with a tumor proportion score (TPS) between 1 and 25%. This threshold could be more challenging to interpret and could have possibly contributed to more variability of the concordance results.

Our study had certain limitations due to its inherent retrospective nature, which limits the causality of associations. The minimum six-month follow-up that allowed for sufficient information on cases may have contributed to overestimating the global PFS by excluding some very aggressive oligometastatic cancers. However, we did assess the impact of potentially confounding factors, including sex and ECOG, which did not have a statistically significant impact in our study group. In addition, the study population included a relatively small proportion of patients with metastasis to other sites than brain, which limits the extent to which conclusions of our study can be applied to those specific patients.

Another limitation was that we did not record all comorbidities. This could have contributed to a certain selection bias, particularly if a patient with multiple comorbidities had less probability of receiving aggressive treatment. If patients with a higher number of comorbidities were indeed overrepresented in Group B, this might have possibly predisposed them to worse survival outcomes. However, patient wishes regarding the choice of aggressive or palliative treatment in oncology are highly variable; therefore, making the impact estimation of this bias hard to predict. On the other hand, this may contribute to the external validity of our study by representing the oncologic clinical reality and by including patients usually excluded from randomized studies. Our results are embedded in an environment in which prospective data is emerging in the field, which will help to characterize the magnitude of the survival benefit in the oligometastatic NSCLC population. Recent long-term results from the SABR-COMET Phase II randomized trial comparing palliative approach and SABR plus palliative approach in oligometastatic cancer of various primary sites including lung cancer, demonstrate a significant absolute five-year OS benefit of 24.6% [21]. Interestingly, the NRG LU002 Phase II/III ongoing trial is studying the progression-free impact of local consolidation with SABR with or without systemic maintenance therapy after initial response to first-line systemic therapy in patients with limited stage IV extracranial disease, which is a slightly different and relevant setting. {NCT03137771 at https://clinicaltrials.gov/}. 

## 5. Conclusions

Our results suggest that serious consideration should be given for a more radical approach when patients present with oligometastatic lung cancer disease. When comparing the management of all stages, patients receiving aggressive treatment at both primary and metastatic sites as well as patients receiving aggressive treatment at primary tumor sites have better mOS and mPFS compared with those treated only at metastatic sites or those receiving palliative treatment. Furthermore, when stratified by “modified” staging, this mOS and mPFS benefit remained significant for stages II and III, but not for stage I. This approach may provide a longer life expectancy and an improved quality of life for those well selected patients who may live up to a year without regular treatment. This may also lead to better resource stewardship for the healthcare system. Importantly, our study is one of the first to demonstrate the negative prognostic impact of PD-L1 expression in oligometastatic NSCLC, illustrating a potential pivotal role of immunotherapy in this group of patients.

## Figures and Tables

**Table 1:** Demographic and disease characteristics of patients.

Characteristics	Brain (*n* = 50) 1 Metastases (*n* = 37) 2 Metastases (*n* = 9) 3 Metastases (*n* = 4)	Bone (*n* = 4)	Adrenal (*n* = 6)	Liver (*n* = 2)	Contralateral Lung (*n* = 5)	Total N (*n* = 67)
**Sex**						
Male	29 (43)	2 (3)	3 (5)	2 (3)	2 (3)	38 (57)
**Age at diagnosis**						
< 65	34 (51)	3 (5)	3 (5)	1 (2)	4 (6)	45 (67)
≥65	16 (24)	1 (2)	3 (5)	1 (2)	1 (2)	22 (33)
**Smoking history**						
Positive	50 (75)	2 (3)	6 (9)	2 (3)	5 (8)	65 (97)
**ECOG**						
0	13 (19)	1 (2)	1 (2)	0 (0)	2 (3)	17 (25)
1	20 (30)	3 (5)	2 (3)	2 (3)	1 (2)	28 (42)
2	5 (8)	0 (0)	0 (0)	0 (0)	0 (0)	5 (8)
3	0 (0)	0 (0)	0 (0)	0 (0)	0 (0)	0 (0)
4	0 (0)	0 (0)	0 (0)	0 (0)	0 (0)	0 (0)
N/A	12 (18)	0 (0)	3 (5)	0 (0)	2 (3)	17 (25)
**Histology**						
Adenocarcinoma	36 (54)	3 (5)	4 (6)	0 (0)	5 (8)	48 (2)
Large Cell Carcinoma	3 (5)	0 (0)	0 (0)	0 (0)	0 (0)	3 (5)
Squamous Cell Carcinoma	2 (3)	0 (0)	2 (3)	2 (3)	0 (0)	6 (9)
Unspecified	9 (13)	1 (2)	0 (0)	0 (0)	0 (0)	10 (15)
PD-L1 status *						
Positive	16 (41)	0 (0)	1 (3)	0 (0)	0 (0)	17 (44)
Negative	18 (46)	2 (5)	0 (0)	0 (0)	2 (5)	22 (56)
Insufficient tissue	16	2	5	2	3	28
***EGFR*** **status**						
Positive	0 (0)	0 (0)	0 (0)	0 (0)	0 (0)	0 (0)
Negative	29 (43)	4 (6)	4 (6)	1 (1)	3 (4)	41 (61)
Insufficient tissue	21 (31)	0 (0)	2 (4)	1 (1)	2 (3)	26 (39)
***ALK*** **status**						
Positive	0 (0)	0 (0)	0 (0)	0 (0)	0 (0)	0 (0)
Negative	35 (52)	4 (6)	5 (7)	1 (1)	4 (6)	49 (73)
Insufficient tissue	15 (22)	0	1 (1)	1 (1)	1 (1)	18 (27)

Number in ( ) represents percentage of cohort (*n* = 67). * Mutation tests for PD-L1 were not performed because testing was not widespread at the time of diagnosis for these patients. Patients with insufficient tissue were excluded from percentage calculation for PD-L1 status and mutational status.ECOG: Eastern Cooperative Oncology Group; N/A: not available; PD-L1: programmed cell death ligand 1; EGFR: Epidermal growth factor receptor; ALK: Anaplastic lymphoma kinase.

**Table 2:** Type of treatment and staging by metastatic site.

Metastatic Site	Brain (*n* = 50)	Bone (*n* = 4)	Adrenal gland (*n* = 6)	Liver (*n* = 2)	Contralateral lung (*n* = 5)	Total (*n* = 67)
**Aggressive treatment**						52 (78)
**Group A**						29 (43)
Metastatic site + Primary site + Adjuvant chemotherapy (Group A1)	4 (6)	1 (2)	0 (0.0)	1 (2)	1 (2)	7 (10)
Metastatic site + Primary sites (Group A2)	21 (31)	0 (0.0)	1 (2)	0 (0)	0 (0)	22 (33)
**Group B1**						4 (6)
Primary site only	1 (2)	0 (0)	0 (0)	0 (0)	0 (0)	1 (2)
Primary site + adjuvant chemotherapy	0 (0)	1 (2)	0 (0)	0 (0)	2 (3)	3 (5)
**Group B2**						19 (28)
Metastatic site only	11 (16)	0 (0)	0 (0)	0 (0)	1 (2)	12 (18)
Metastatic site + palliative chemotherapy +/− palliative RT	6 (9)	1 (2)	0 (0)	0 (0)	0 (0)	7 (10)
**Palliative treatment (Group B3)**						15 (22)
Watch & Wait	0 (0)	0 (0)	2 (3)	0 (0)	0 (0)	2 (3)
Palliative chemotherapy	0 (0)	0 (0)	2 (3)	1 (2)	1 (2)	4 (6)
Palliative tyrosine-kinase inhibitor	0 (0)	1 (2)	0 (0)	0 (0)	0 (0)	1 (2)
Palliative chemotherapy + RT	1 (2)	0 (0)	0 (0)	0 (0)	0 (0)	1 (2)
Palliative RT	6 (9)	0 (0)	1 (2)	0 (0)	0 (0)	7 (10)
**Staging by metastatic site**						
**8^th^ IASLC classification TN staging** (*p* = 0.053)						
T1–4 N0	22 (33)	2 (3)	2 (3)	2 (3)	3 (5)	31(46)
T1–4 N1	3 (5)	0 (0)	0 (0)	0 (0)	1 (2)	4 (6)
T1–4 N2	17 (25)	2 (3)	3 (5)	0 (0)	1 (2)	23 (34)
T1–4 N3	8 (12)	0 (0)	1 (2)	0 (0)	0 (0)	9 (13)
**“Modified” Stage** based on the combined 8^th^ IASLC Edition T and N stages (*p* = 0.142)						
Stage I (T1–2 N0 M1b)	13 (20)	2 (3)	0 (0)	1 (2)	2 (3)	18 (27)
Stage II (T1–2 N1 M1b and T3–4 N0 M1b)	10 (15)	0 (0)	0 (0)	1 (2)	2 (3)	13 (19)
Stage III (T1–2 N2 M1b and T3–4 N1 M1b)	27 (40)	2 (3)	6 (9)	0 (0)	1 (2)	36 (54)

Number in ( ) represents percentage of cohort (n=67); RT: radiation therapy; IASLC: International Association for the Study of Lung Cancer.

**Table 3:** PD-L1 status and clinical characteristics of subgroup of 39 patients.

Demographic and Tumoral Characteristics	Subgroup	PD-L1 Positive (≥25%) *n* (%)	PD-L1 Negative (<25%) *n* (%)	*p*-value
Sex	Men	8 (21)	13 (33)	0.455
	Women	9 (23)	9 (23)	
Age at diagnosis	≥50 y.o.	15 (38)	19 (49)	0.862
	<50 y.o.	2 (5)	3 (8)	
Smoking history	≤25 pack-years	2 (5)	2 (8)	0.371
	>25 to <50 pack-years	8 (21)	14 (36)	
	≥50 pack-years	5 (13)	6 (15)	
	N/A	2 (5)	0	
ECOG at diagnosis	0	3 (8)	7 (18)	0.719
	1	6 (15)	8 (21)	
	2	1 (3)	1 (3)	
	N/A	7 (18)	6 (15)	
T staging	1	1 (3)	9 (23)	0.065
	2	6 (15)	8 (21)	
	3	4 (10)	2 (5)	
	4	4 (10)	2 (5)	
	N/A	2 (5)	1 (3)	
N staging	0	8 (21)	15 (38)	0.470
	1	1 (3)	1 (3)	
	2	5 (13)	5 (13)	
	3	3 (8)	1 (3)	
Histology	Adenocarcinoma	11 (28)	17 (44)	0.849
	Squamous Cell Carcinoma	1 (3)	1 (3)	
	Large Cell Carcinoma	1 (3)	1 (3)	
	Poorly Differentiated	4 (10)	3 (8)	

y.o.: years old, N/A: not available

## Data Availability

The data presented in this study are available on request from the corresponding author. The data are not publicly available due to privacy and ethical reasons.

## Supplementary Materials

The following are available online at https://www.mdpi.com/1718-7729/28/1/59/s1, Table S1: Treatment type and demographic characteristics of patients, Table S2: Survival of patients with aggressive or non-aggressive treatment classified by “Modified” Tumor Stage, Table S3: PD-L1 status and tumor site of subgroup of 39 patients, Table S4: PD-L1 concordance between primary and metastatic tissues in 10 patients, Figure S1: Overall survival based on the number of brain metastasis, Figure S2: Overall survival comparison between four different therapeutic approaches, Figure S3: Overall survival of 39 patients stratified by treatment of primary site and PD-L1 status, Figure S4: Overall survival of 39 patients stratified by treatment of metastatic site and PD-L1 status.
