# Supplementary material for: Survival Impact of Aggressive Treatment and PD-L1 Expression in Oligometastatic NSCLC
Source: Curr Oncol. 2021 Jan 20;28(1):593–605. doi: 10.3390/curroncol28010059 (PMC7924378; doi:10.3390/curroncol28010059)
Supplement: Supplementary file 1 [file curroncol-28-00059-s001.pdf]

Supplementary Materials: Survival Impact of Aggressive Treatment and PD-L1 Expression in Oligometastatic NSCLC

Camille Gauvin, Vimal Krishnan, Imane Kaci, Danh Tran-Thanh, Karine Bédard, Roula Albadine, Charles Leduc, Louis Gaboury, Normand Blais, Mustapha Tehfe, Bertrand Routy and Marie Florescu

Table 1. Treatment type and demographic characteristics of patients.

Demographic characteristics	Treatment			
	**Aggressive at both sites (Group A) n=29 (%)**	**Primary site only (Group B1) n=4 (%)**	**Metastatic site only (Group B2) n=19 (%)**	**Palliative (Group B3) n=15 (%)**
**Sex**				
Male	15 (52)	0 (0)	13 (68)	10 (67)
**Smoking history**				
Positive	29 (100)	3 (75)	19 (100)	14 (93)
**Age at diagnosis**				
<65	23 (79)	3 (75)	13 (8)	6 (40)
≥65	6 (21)	1 (25)	6 (32)	9 (60)
**ECOG Score**				
0	10 (35)	1 (25)	3 (16)	3 (20)
1	12 (41)	2 (50)	8 (42)	6 (40)
2	1(4)	0 (0)	4 (21)	0 (0)
3	0 (0)	0 (0)	0 (0)	0 (0)
4	0 (0)	0 (0)	0 (0)	0 (0)
N/A	6 (21)	1 (25)	4 (21)	6 (40)

ECOG: Eastern Cooperative Oncology Group; N/A: not applicable.

Table S2. Survival of patients with aggressive or non-aggressive treatment classified by "Modified" Tumor Stage.

8th IASLC Classification	Aggressive at Both Primary and Metastatic Sites *n* = 29 (%)	Non-Aggressive Treatments *n* = 38 (%)	*p*-value
**Stage I, *n* = 18**			
n	9 (31)	9 (24)	
OS (m)	42	16	0.316
PFS (m)	12	12	0.669
**Stage II, *n* = 13**			
n	7 (24)	6 (16)	
OS (m)	34	6	0.024
PFS (m)	Not reached	6	0.016
**Stage III, *n* = 36**			
n	13 (45)	23 (60)	
OS (m)	22	4	0.001
PFS (m)	19	3	0.003

"Modified" Tumor Stage: refers to the combined stage T and N of the IASLC staging eighth edition; OS: overall survival; PFS: progression-free survival; (m): months.

Table 3. PD-L1 status and tumor site of subgroup of 39 patients.

PD-L1 Status	Lung Primary *n* (%)	Metastasis *n* (%)
Positive (≥25%)	12 (55)	8 (30)
Negative (<25%)	10 (45)	19 (70)
Insufficient tissue	17	12

Table 4. PD-L1 concordance between primary and metastatic tissues in 10 patients.

Patient	Age	Sex	Progression-Free Survival (months)	Overall Survival (months)	PD-L1 (Primary Site)	PD-L1 (Metastasis)	Concordance
1	73	F	55	55	-	+	No
2	54	F	19	52	+	-	No
3	54	M	4	4	+	+	Yes
4	47	M	5.6	32	-	-	Yes
5	47	F	43	68	-	-	Yes
6	46	F	3.7	20	-	-	Yes
7	46	F	6	15	+	+	Yes
8	69	M	17.5	20	-	-	Yes
9	56	M	7.2	16	-	-	Yes
10	59	M	32	36	+	+	Yes
Median	54	-	12.4	26	-	-	80% (8/10)

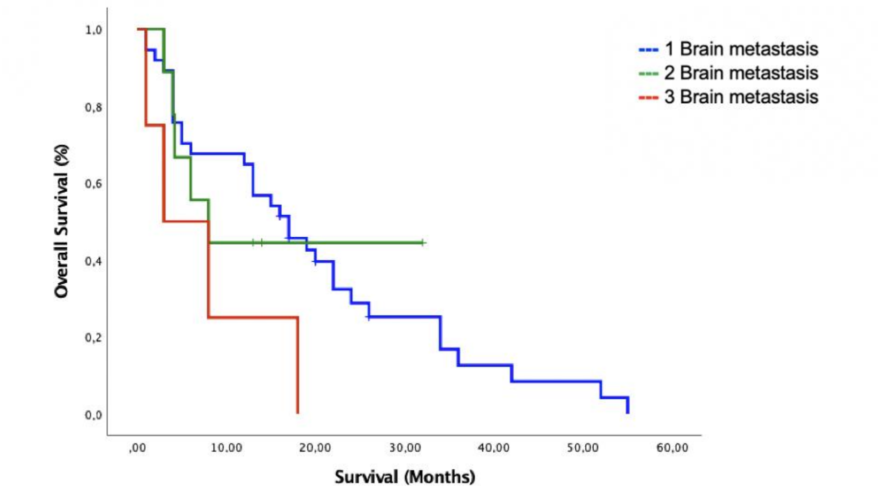

Number of Brain Metastasis	*n* (%)<br>*n* total = 50	Median OS (months)	OS 1 year (%)	OS 2 year (%)
1	37 (74)	17	65	29
2	9 (18)	8	44	44
3	4 (8)	3	25	0
p-value = 0.121				

Figure S1. Overall survival based on the number of brain metastasis.

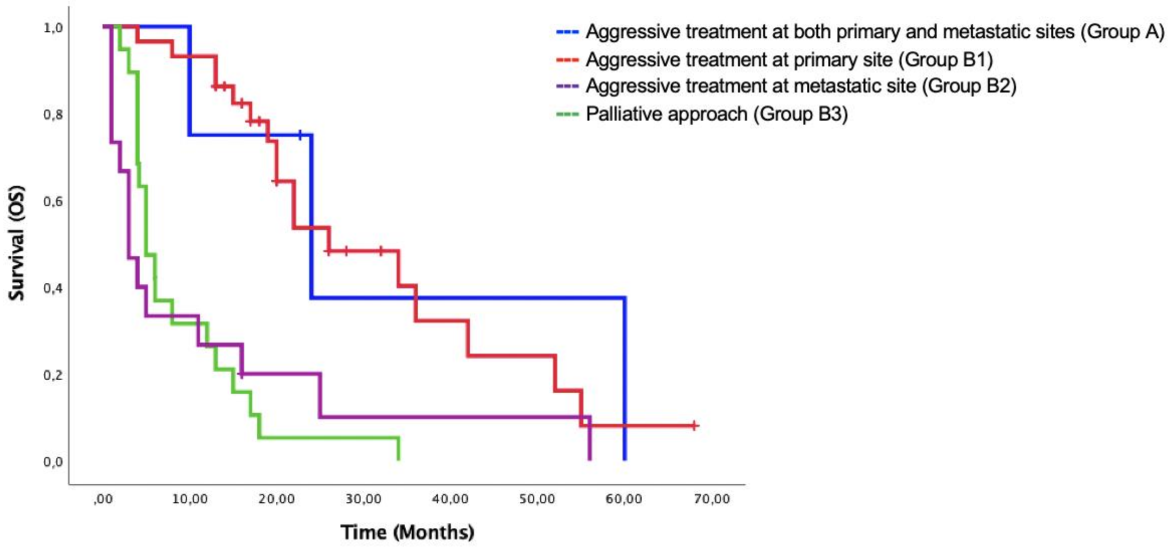

Treatment	*n* =67 (%)	mOS (months)	OS 2 years (%)
Aggressive treatment at both primary and metastatic sites (Group A)	29 (43)	26	54
Aggressive treatment at primary site (Group B1)	4 (6)	24	38
Aggressive treatment at metastatic site (Group B2)	19 (28)	5	5
Palliative approach (Group B3)	15 (22)	3	20
***p*-value**= 0.0001			

Figure S2. Overall survival comparison between four different therapeutic approaches.

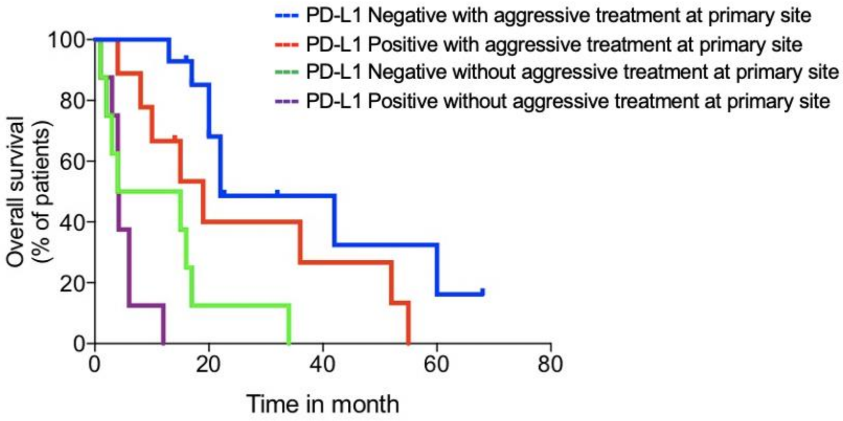

Treatment	mOS (months)	mPFS (months)
**PD-L1 Negative (*n* = 22)**		
Curative Tx (*n* = 14)	22	12.2
Palliative Tx (*n* = 8)	4	4
**PD-L1 Positive (*n* = 17)**		
Curative Tx (*n* = 9)	15	9.6
Palliative Tx (*n* = 8)	4	3
***p*-value**	0.04	0.22

Figure S3. Overall survival of 39 patients stratified by treatment of primary site and PD-L1 status. Kaplan-Meier overall survival analysis of patients with PD-L1 status available comparing PD-L1 status and treatment approach at primary site.

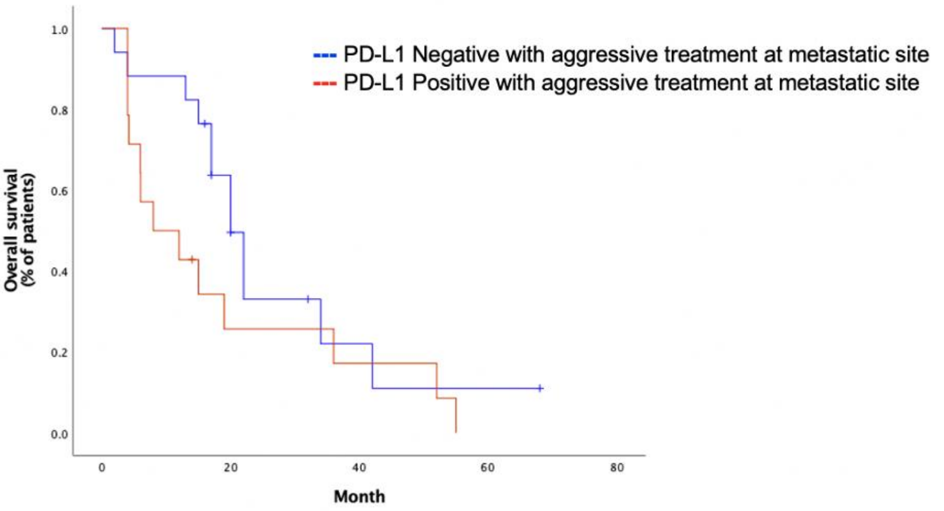

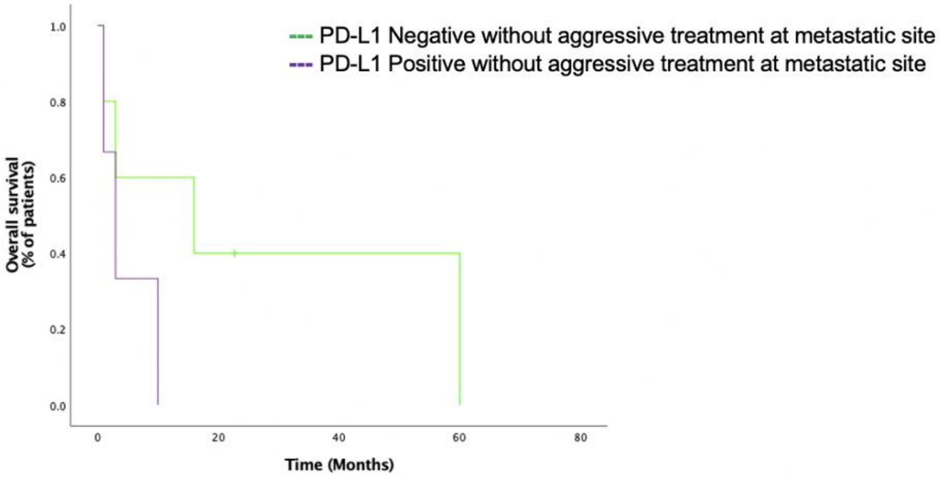

Treatment	mOS (months)	mPFS (months)
**PD-L1 Negative (*n* = 22)**		
**Curative Tx (*n* = 17)**	20.0	12.2
**Palliative Tx (*n* = 5)**	16.0	14.7
**PD-L1 Positive (*n* = 17)**		
**Curative Tx (*n* = 14)**	8.0	6.0
**Palliative Tx (*n* = 3)**	3.0	3.0
***p*-value**	0.07	0.15

Figure S4. Overall survival of 39 patients stratified by treatment of metastatic site and PD-L1 status. Kaplan-Meier overall survival analysis of patients with PD-L1 status available comparing PD-L1 status and treatment approach at metastatic site.

Publisher's Note: MDPI stays neutral with regard to jurisdictional claims in published maps and institutional affiliations.

© 2020 by the authors. Licensee MDPI, Basel, Switzerland. This article is an open access article distributed under the terms and conditions of the Creative Commons Attribution (CC BY) license (http://creativecommons.org/licenses/by/4.0/).
